# Effect of Monomers of 3-Hydroxyhexanoate on Properties of Copolymers Poly(3-Hydroxybutyrate-*co* 3-Hydroxyhexanoate)

**DOI:** 10.3390/polym15132890

**Published:** 2023-06-29

**Authors:** Tatiana G. Volova, Mayya V. Uspenskaya, Evgeniy G. Kiselev, Aleksey G. Sukovatyi, Natalia O. Zhila, Aleksander D. Vasiliev, Ekaterina I. Shishatskaya

**Affiliations:** 1 Institute of Biophysics SB RAS, Federal Research Center “Krasnoyarsk Science Center SB RAS”, Akademgorodok 50/50, 660036 Krasnoyarsk, Russia; volova45@mail.ru (T.G.V.); evgeniygek@gmail.com (E.G.K.); a.sukovatiy@yandex.ru (A.G.S.); shishatskaya@inbox.ru (E.I.S.); 2 School of Fundamental Biology and Biotechnology, Siberian Federal University, Svobodnyi Av. 79, 660041 Krasnoyarsk, Russia; 3 Chemical Engineering Center, Research Institute «Bioengineering» ITMO University, Kronverksky Pr. 49, 197101 Saint Petersburg, Russia; mv_uspenskaya@itmo.ru; 4 V. Kirensky Institute of Physics SB RAS, Federal Research Center “Krasnoyarsk Science Center SB RAS”, Akademgorodok 50/38, 660036 Krasnoyarsk, Russia; adva@iph.krasn.ru; 5 Basic Department of Solid State Physics and Nanotechnology, School of Engineering Physics and Radio Electronics, Siberian Federal University, Kirensky St. 26, 660074 Krasnoyarsk, Russia

**Keywords:** P(3HB-*co*-3HHx) copolymers, P(3HB) homopolymer, various synthesis conditions, molecular weight, crystallinity, thermal properties, isothermal crystallization, spherulites

## Abstract

The properties of poly(3-hydroxybutyrate-*co*-3-hydroxyhexanoate) P(3HB-*co*-3HHx) copolymers with different ratios of monomers synthesized by the wild-type strain *Cupriavidus necator* B-10646 on sugars, and an industrial sample from Kaneka synthesized by the recombinant strain *C. necator* NSDG-ΔfadB1 on soybean oil, were studied in a comparative aspect and in relation to poly(3-hydroxybutyrate) P(3HB). The copolymer samples, regardless of the synthesis conditions or the ratio of monomers, had reduced values of crystallinity degree (50–60%) and weight average molecular weight (415–520 kDa), and increased values of polydispersity (2.8–4.3) compared to P(3HB) (70–76%, 720 kDa, and 2.2). The industrial sample had differences in its thermal behavior, including a lower glass transition temperature (−2.4 °C), two peaks in its crystallization and melting regions, a lower melting point (T_melt_) (112/141 °C), and a more pronounced gap between T_melt_ and the temperature of thermal degradation (T_degr_). The process, shape, and size of the spherulites formed during the isothermal crystallization of P(3HB) and P(3HB-*co*-3HHx) were generally similar, but differed in the maximum growth rate of the spherulites during exothermic crystallization, which was 3.5–3.7 μm/min for P(3HB), and 0.06–1.25 for the P(3HB-*co*-3HHx) samples. The results from studying the thermal properties and the crystallization mechanism of P(3HB-*co*-3HHx) copolymers are important for improving the technologies for processing polymer products from melts.

## 1. Introduction

The potential for the use of living systems in biotechnological processes could provide a wide range of materials and products necessary for human life, including for food and medical and technical purposes, by using carbon-containing compounds of various origins as raw materials. The expansion of the spectrum of substrates observed today, including the use of waste and renewable natural raw materials for biosynthetic processes, enhances the contribution of biotechnology to the circular economy and increases the value of biotechnological products [1,2]. This situation raises the potential for microbial polymers polyhydroxyalkanoates (PHAs), the so-called “green” plastics, to be an alternative to and to gradually replace non-degradable synthetic oil-derived polyolefins, whose accumulation in the biosphere has created a global environmental problem. World production of synthetic plastics has reached 400 million tons per year; it is expected that by 2050 it will have increased to 1.0 Gt per year [3].

PHAs are a family of biodegradable thermoplastic polymers of various chemical structures with different physicochemical properties [4,5,6,7,8,9,10,11]. These biopolymers are considered to be the most promising material of the 21st century, and have a high potential for various applications, from tissue engineering, reconstructive medicine, and modern pharmacology to municipal and agricultural purposes [12,13,14,15,16,17]. PHAs have great potential to contribute to “The Circular Economy” [16,17]. This is due to the fact that C substrates, which have different degrees of reduction and cost, can be used as raw materials for the synthesis of PHAs, including both individual compounds as well as wastes of various origins (vegetable raw materials, food, pharmaceutical, alcohol, pulp and paper, industrial, etc.). Therefore, PHAs are called “Circular Materials for Sustainable Development and Growth” [11,18,19,20]. The ability to use waste for the production of PHAs is becoming an increasingly important contribution of biotechnology toward solving the problem of reducing the amount of waste in the biosphere, as well as for increasing the efficiency of industrial production along the path of the circular economy.

The most common and studied of the PHA family is 3-hydroxybutyric acid homopolymer (poly(3-hydroxybutyrate)), P(3HB), which is characterized by its high crystallinity and hydrophobicity, as well as the fact that it does not crystallize with the formation of an ordered structure. Therefore, polymer products from P(3HB) do not have elasticity and become brittle with time [21,22,23]. The properties of P(3HB) can be improved using biological, chemical, and physical methods, such as obtaining composites of P(3HB) with other materials, through chemical modification, the physical treatment of the surface of products derived from it with plasma or laser cutting, as well as the synthesis of copolymers with a different set and ratio of monomers [24,25,26,27,28]. These methods allow positive changes in the properties of PHAs, including a decrease in crystallinity, an increase in the flexibility and mechanical strength of its products, hydrophilicity, adhesive properties of the surface, etc.

Particularly promising PHAs are copolymers with a low crystallinity and the properties of elastomers. These are short-chain copolymers of 3- and 4-hydroxybutyrate [P(3HB-*co*-4HB)], as well as medium-chain PHAs containing 3-hydroxyhexanoate (3HHx) monomers [5,6,7]. The random copolymer P(3HB-*co*-3HHx) shows a broader processing window than the short chain P(3HB) and poly(3-hydroxybutyrate-*co*-3-hydroxyvalerate) P(3HB-*co*-3HV). Moreover, P(3HB-*co*-3Hx) has good thermo-mechanical and physicochemical properties, due to its tailorable composition of elastomeric (3HHx) and highly crystalline (3HB) monomers. Therefore, this type of PHA is more suitable for producing elastic products for tissue engineering, as well as packaging products obtained by melt extrusion [14,29,30,31]. For instance, the incorporation of monomers such as 3HHx into P(3HB) results in the P(3HB-*co*-3HHx) copolymer, which has elastomeric properties, such as high elasticity, low crystallinity, and high elongation at break. The flexibility of this copolymer can be varied depending on the 3HHx molar content [32].

However, the synthesis of P(3HB-*co*-3HHx) copolymers with high inclusions of 3HHx monomers is a very difficult biotechnological task since, as a rule, their production requires the introduction of additional carbon sources as precursor substrates, which usually inhibit the growth of microorganisms. This negatively affects the overall productivity of the biosynthesis process in terms of cell biomass and total PHA yields [33]. The possibility of the productive synthesis of PHA-containing 3-hydroxyhexanoate monomers is additionally complicated by the specifics of the metabolism of hexanoate as a precursor substrate, since it is metabolized in the fatty acid β-oxidation cycle into shorter fragments (C4 + C2), thereby reducing the amount of substrate for the formation and incorporation of 3HHx monomers into the polymer chain. To obtain high yields of PHAs with a high content of medium-chain monomers, it is necessary to organize special conditions under which the reactions of the β-oxidation of hexanoate are blocked; the second possibility is the production of genetically engineered strains with altered biochemical pathways for the synthesis of 3HHx monomers [9,14,31].

The industrial production of P(3HB-*co*-3HHx) copolymers has been mastered by large PHA manufacturers, such as Kaneka Corporation (KITA-KU Osaka, Japan), which produces these copolymers under the Green Planet™ trademark [34]. The company Procter & Gamble, Chemicals (Cincinnati, OH, USA) produces copolymers under the brand name NodaxTM [35]. Copolymers are also produced by Danimer Scientific (Winchester, KY, United States) [36] and Bluepha^®^ for the blue planet (Beijing, China) [37]. Numerous scientific teams who have technologies for the synthesis of P(3HB-*co*-3HHx) copolymers use various strains, various substrates, and have developed their own fermentation technologies for this purpose. Therefore, the technologies differ in the production indicators, economic efficiency, and properties of the P(3HB-*co*-3HHx) copolymers obtained.

This paper presents the results of changes in the structural features and physicochemical characteristics of P(3HB) homopolymer when 3-hydroxyhexanoate monomers are included in its C-chain. This is based on the results of the biological synthesis of the copolymers of P(3HB-*co*-3HHx) and a comparative study of these copolymers with those produced on an industrial scale by Kaneka Corporation (Osaka, Japan), and laboratory samples synthesized at the Institute of Biophysics of the Siberian Branch of the Russian Academy of Sciences (Moscow, Russia), which differ in the ratio of monomers.

## 2. Materials and Methods

### 2.1. Materials

Samples of polyhydroxyalkanoates (PHAs) from various sources were studied. The commercial sample of P(3HB-*co*-3HHx) copolymer with 11 mol.% of 3-hydroxyhexanoate (3HHx) monomers, was synthesized by a recombinant strain of *Cupriavidus necator* NSDG-ΔfadB1 using a patented technology [38] (trademark of Green Planet™) purchased from Kaneka Corporation (KITA-KU Osaka, Japan). Laboratory samples are represented by P(3HB-*co*-3HHx) copolymers with 9.0, 16.4, and 38.0 mol.% of 3-hydroxyhexanoate (3HHx) monomers and 3-hydroxybutyrate [P(3HB)] homopolymers, which were synthesized by wild-type strains of *Cupriavidus necator* B-10646 [39] and *Alcaligenes eutrophus* B-5786 (renamed to *Ralstonia eutropha*—*Cupriavidus necator*) according to directions from the authors and patented technology (trademark Bioplastotan) [40,41].

### 2.2. PHA Recovery from Cell Biomass

The polymer was extracted from the cell biomass with dichloromethane; the resulting extract was concentrated on an R/210V rotary evaporator (Büchi, Flawil, Switzerland) and then precipitated with ethanol. Repeating the procedures of polymer dissolution and reprecipitation ensured the removal of impurities and the obtaining of homogeneous samples. The chemical purity of the samples was tested using a 7890A gas chromatograph equipped with a 5975C chromatograph-mass spectrometer (Agilent Technologies, Santa Clara, CA, USA). The polymer was dried in a fume hood at room temperature for 72 h.

### 2.3. PHA Chemical Composition

The purity of the PHA and its chemical content was determined with chromatography of the methyl esters of the fatty acids after methanolysis of the purified polymer samples, using a 7890A chromatograph-mass spectrometer (Agilent Technologies, Santa Clara, CA, USA) equipped with a 5975C mass detector (Agilent Technologies, Santa Clara, CA, USA) [42].

### 2.4. NMR and IR Spectroscopy

The molecular structure of the PHA was studied with NMR methods using a Varian VXP-500 S spectrometer with ^1^H NMR. PHA samples were analyzed using FTIR spectroscopy. IR spectra were taken in the 400–4000 cm^−1^ range using a “NICOLET 6700” FT-IR spectrometer (Thermo Scientific, Waltham, MA, USA) and a Smart Orbit accessory, using the attenuated total reflection (ATR) technique.

### 2.5. Physicochemical Properties of PHA

The physicochemical properties of the PHA were examined using high-performance liquid chromatography, X-ray structure analysis, and differential scanning calorimetry. All methods have been previously described in detail [43].

The molecular weight and molecular weight distribution of the PHA specimens were examined using a size-exclusion chromatography (Agilent Technologies 1260 Infinity, Waldbronn, Germany) equipped with a DB-35MS column. Molecular weights (weight average, Mw, and number average, Mn) and polydispersity (Ð = Mw/Mn) were determined.

To determine the thermal properties of the PHA samples, thermal analysis was carried out employing a DSC-1 differential scanning calorimeter (Mettler Toledo, Schwerzenbac, Switzerland) and TGA (Mettler Toledo, Schwerzenbac, Switzerland). The crystallization temperature (T_cryst_) was detected by exothermic peaks, and the glass transition temperature (T_g_), melting point (T_melt_), and thermal degradation temperature (T_degr_) were determined by the endothermic peaks on the thermograms. The thermograms were analyzed using “STARe v11.0” software (Mettler Toledo, Schwerzenbac, Switzerland).

An X-ray diffraction analysis using a D8 ADVANCE X-ray powder diffractometer with a VANTEC fast linear detector (Bruker AXS, Karlsruhe, Germany) was carried out to determine the crystallinity of the PHA. The first step was to subtract the instrumental background of the radiograph. Crystallinity degree (C_x_) was detected as the ratio of the area under the radiograph with the subtracted amorphous background to the area without subtracting the background. The Eva program from the diffractometer software application was applied for the calculations.

### 2.6. Optical Studies of the Formation of Spherulites

The morphology and radial growth rate of the spherulites of the PHA samples subjected to isothermal crystallization at different temperatures were observed using a polarizing optical microscope (POM) (Nikon, Eclipse E600 POL, Tokyo, Japan) equipped with a heating stage (hotstage) (Linkam LTS420, Redhill, UK). The samples were first melted at 195 °C for 3 min to destroy the thermal prehistory, and then cooled to the desired (studied) crystallization temperature. The growth rate of the spherulites (G) was calculated by measuring the radius, R, as a function of crystallization time, t, and expressed in µm/min. The observation was continued until the field of view was completely covered by spherulites.

## 3. Results and Discussion

To reveal how the properties of P(3HB) change when medium-chain monomers of 3-hydroxyhexanoate (3HHx) are included in the 3-hydroxybutyrate (3HB) chain, the properties of P(3HB-*co*-3HHx) copolymers with different ratios of monomers synthesized with various technologies on various carbon substrates by recombinant and natural strains were studied.

### 3.1. Characteristics of Poly(3-Hydroxybutyrate)

**Figure 1:**
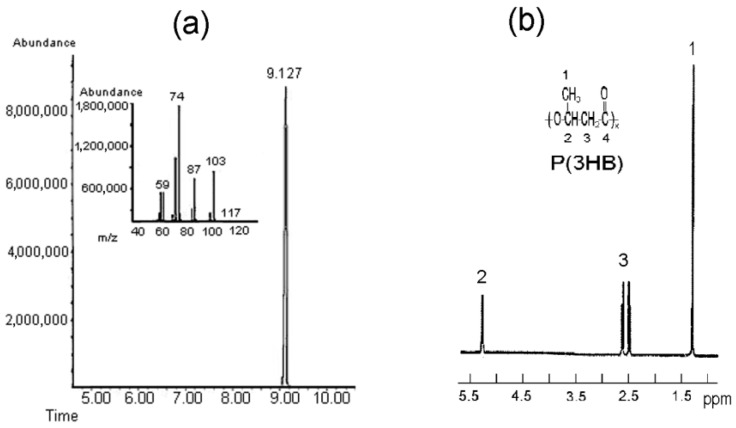
Ion chromatograms and mass spectra of the methyl ester of 3-hydroxybutyrate (**a**) and ^1^H NMR spectrum (**b**) of P(3HB) synthesized by *Cupriavidus necator* B-10646. Figure 1a is taken from reference [43] with permission from Elsevier.

**Figure 2:**
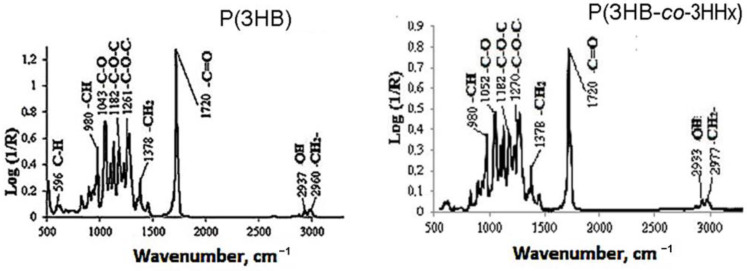
IR spectra of P(3HB) and P(3HB-*co*-3HHx) synthesized by *Cupriavidus necator* B-10646. Taken from reference [43] with permission from Elsevier.

Poly(3-hydroxybutyrate) is an isotactic polyester with regular, identically oriented (“head-to-tail”) successive units of D-β-hydroxybutyric acid: [-O-CH(CH_3_)-CH_2_-CO-]_n_ [44]. The ion chromatogram with mass spectrum and ^1^H NMR spectrum of poly(3-hydroxybutyrate) are given in Figure 1. To reveal the structural features of P(3HB), IR spectroscopy was used, which is used to study the structure of various macromolecules. From the shape of the intensity of the bands in the low-frequency region, it is possible to obtain information about the ratio of ordered and disordered phases. Figure 2 shows the IR spectrum of P(3HB) taken in the range of 400–4000 cm^−1^.

The IR absorption spectra of P(3HB) contain absorption bands corresponding to the vibrations of the main structural units of this polymer, in addition to the absorption bands of the vibrations of the terminal C-OH and COOH groups. Bands of ordered optical densities (crystalline phase) lie in the region of 1261 cm^−1^; the disordered (amorphous phase) are shifted to 1182 cm^−1^. Absorption bands of the asymmetric stretching vibrations of the CH_3_ and CH_2_ groups (2978 and 2960 cm^−1^) were recorded, as were the symmetric stretching vibrations of the CH- and CH_2_-groups (2994 and 2937 cm^−1^), the stretching vibrations of the C=O carbonyl groups, both conjugate (1687 cm^−1^) and non-conjugated (1720 cm^−1^), skeletal CH (599 cm^−1^), and CH deformed groups (622 cm^−1^).

The molecular structure of P(3HB) was previously studied with a high-resolution NMR. It was shown in [45] that the ^13^C (125 MHz) and ^1^H (500 MHz) NMR spectra synthesized by *Alcaligenes eutrophus* B-5786 (this systematic name was subsequently transformed into *Cupriavidus necator*), when carbon dioxide was used as the C substrate, coincided with a high degree of accuracy with spectra taken of P(3HB) samples synthesized by other producers: *A. eutrophus* H16 and *Bacillus megaterium KM*, cultivated on fructose and glucose, respectively [46,47]. An analysis of the constant–dipole interaction between HA, HB, and HX protons in a magnetically isolated fragment made it possible to obtain data averaged over the three possible conformations. Taking into account the generally accepted theoretical values for the constants characterizing the trans and gauche conformations [48], evidence was obtained for the dominance of type I and II conformations in P(3HB) and the practical absence of the type III conformation.

The presence of dynamic processes in samples of solid P(3HB) was confirmed in [49], in which ^1^H NMR spectra were recorded in the temperature range from liquid nitrogen to room temperature [49]. At low temperatures, the proton magnetic resonance (PMR) spectrum of the polymer is a single bell-shaped line about 10 erg wide. Such a line is typical for polymers containing CH_−_, CH_2−_, and CH_3−_proton groups, and indicates the absence of proton mobility with frequencies exceeding 10 kHz [50]. At 125 K, a narrow (less than 1 Oe wide) component appeared in the spectrum, the intensity of which rapidly increased with increasing temperature, reaching 10% of the total intensity. A part of the molecular fragments of the polymer in the transition region (123–143 K) is characterized by intense mobility; the beginning of the narrowing of the spectra testifies to this.

It should be noted that the high proton mobility found in P(3HB) at low temperatures is characteristic of fresh samples. The aging of the polymer (our observations cover a period of about 8 years) [51] is accompanied by a decrease in mobility and a significant (up to 150 K) increase in the temperature at which protons begin to move. In this case, however, the velocities of the particles of the molecular fragments exceeded 10 kHz. This noted effect is probably related to the crystallization of the polymer. Thus, the degree of crystallinity of the fresh samples was about 72 ± 4%; in the “old” samples (long-term storage) it increased to 90% or more.

The degree of crystallinity, which is the ratio of ordered (crystalline) and disordered (amorphous) phases, is one of the most important characteristics of polymeric materials. The ability of P(3HB) and other representatives of PHAs to crystallize is determined by the internal properties of its chains and is characterized by the crystallization temperature (T_cryst_). In PHAs, similarly to many other polymers, crystallization is carried out, but does not capture the entire volume of the material; therefore, these polymers are semi-crystalline objects.

Poly(3-hydroxybutyrate) is a semi-crystalline hydrophobic substance, in which the density of the amorphous phase is 1.177 g/cm^3^, and that of the crystalline phase is 1.23–1.26 g/cm^3^ [52,53]. In P(3HB), the crystalline phase dominates over the amorphous one. This isotactic biopolymer is structurally similar to isotactic polypropylene. The pendant methyl groups of both polymers are attached and oriented in the polymer chain in a single conformation [52]. Poly(3-hydroxybutyrate) is represented by densely packed double helices, repeating at a distance of 5.95 Å, and twisting twice to the right around the axis. The helix conformation is stabilized by the interaction of carbonyl–methyl groups and does not depend on hydroxyl groups. The P(3HB) chain has 2_1_ helical conformations. The orthorhombic cells in the lattice at the P2_1_2_1_2_1_ group space are characterized by the following parameters: a = 5.76 Å, b = 13.20 Å, and c = 5.96 Å [54]. The stability of the P(3HB) cell parameters and their independence from heating and the crystallization process during melt cooling were shown in [30]. X-ray diffraction patterns of P(3HB) fibers showed a repetition along the chain axis at a distance of 0.596 µm of densely packed double antiparallel chains packed into orthorhombic lattice cells [55,56,57].

**Figure 3:**
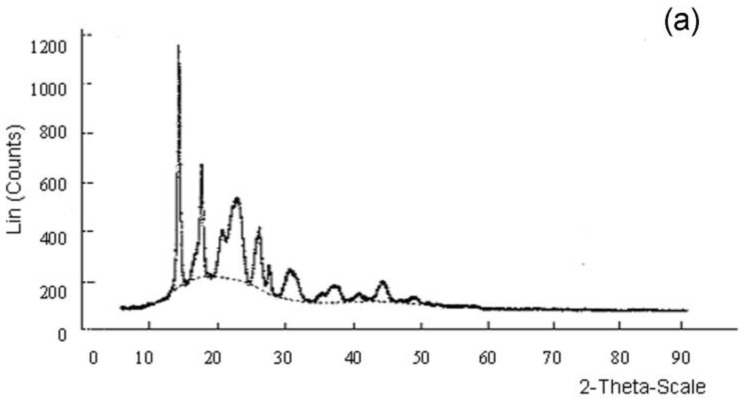

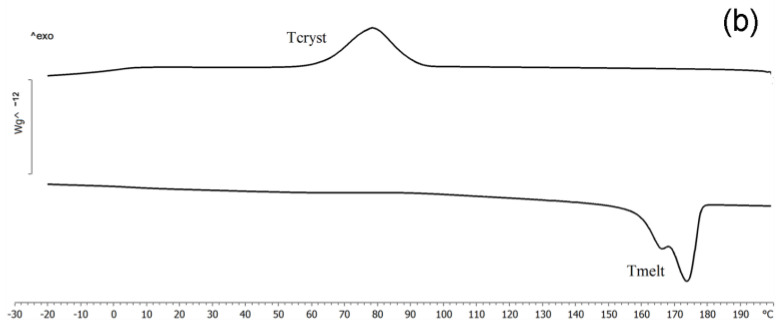
X-ray spectrum (**a**) and thermogram (**b**) of P(3HB) synthesized by *Cupriavidus necator* B-10646.

The results of X-ray studies of poly(3-hydroxybutyrate) synthesized by *Cupriavidus necator* B-10646 are shown in Figure 3a. P(3HB) is represented by two phases—amorphous and crystalline; in this case, the ordered phase dominates over the amorphous one. To determine the degree of crystallinity P(3HB), spectra were taken in the range of angles 5° ≤ 2θ ≤ 60°; the resulting curve R(s_p_^2^) was approximated by a straight line. The samples were scanned in this range of angles, with a step of 0.04° and an 8 s exposure, to measure the intensity at a point, 2θ = 23°. Figure 3a shows the X-ray spectrum of the P(3HB) sample, which has a degree of crystallinity (C_x_) of 70%. The scatter of the values obtained from a representative series of P(3HB) samples lies in the range of 68–76%.

The molecular weight distribution, including the values of weight average (M_w_), number average (M_n_) molecular weight, and polydispersity (Ð), are among the basic characteristics of macromolecular compounds. The molecular weight of P(3HB), as well as other types of PHAs, is a variable parameter that significantly depends on the mode and duration of fermentation, as well as on the procedure for extracting the polymer from the bacterial biomass and removing lipid impurities. Therefore, data on the molecular weight of P(3HB) can vary by an order of magnitude according to different authors [6,58]. The method which we used for isolating and purifying the polymer, using detergents in combination with organic solvents [59], obtains chemically pure homogeneous samples with a weight average molecular weight (M_w_) from 500–700 to 800–1000 kDa at close polydispersity values, from 1.5 up to 2.5. An analysis of the published data shows that the molecular weight of P(3HB) can range from several hundreds to millions of Da. This value depends on the type of producer used, the conditions of its cultivation, as well as the method for the extraction of the polymer from the biomass and the solvents used [60,61]. Considerable attention has been paid to this parameter. It is known that the mechanical strength of P(3HB) decreases significantly if its molecular weight is less than 400 kDa, so at low values of this parameter (M_w_ 200 kDa) the polymer is very brittle [62].

The temperature characteristics of PHAs and their ability to crystallize in the native state are significant parameters since they determine the thermomechanical properties and, consequently, the possibility of processing these polymers into products from melts. The thermal properties of P(3HB) have been actively investigated by several researchers [63,64,65]. The temperature at which P(3HB) undergoes deformation is somewhat lower than the temperature of thermal degradation; therefore, the gas state in polymers is not realized, and the main type of phase equilibrium in them is the condensed state—crystalline, glassy, viscous, and liquid [52,66]. The temperature dynamics of successive phase transitions has been studied for P(3HB) using the DTA method in a number of works [67,68,69]. On thermograms at temperatures of −10 °C < T < 10 °C, a transition peak from the glassy state to a more mobile amorphous state was identified. The next peak was recorded in the region of +50 °C, this is the crystallization peak, and then the peak at the melting point follows. It has been shown that for homogeneous P(3HB), the melting point lies in the range of 176–180 °C; the temperature at the beginning of crystallization is in the region of 47 °C.

The temperature characteristics of poly(3-hydroxybutyrate) synthesized by the *Cupriavidus necator* B-10646 strain on CO_2_ or sugars as a carbon source have been studied using DSC and TGA (Figure 3b). The glass transition temperature (T_g_) for the polymers of this class, according to various sources, is in a range from 0 to 10 °C. The glass transition temperature of the synthesized sample was 5 °C. Poly(3-hydroxybutyrate) has a high degree of crystallinity; therefore, the glass transition effect on thermograms is poorly pronounced, in contrast to copolymer PHAs. The melting point of the test sample was 174 °C after the first heating. The actual melting point (T_melt_), obtained as a result of the repeated heating of the sample, was 173 °C. In this case, the melting point may vary somewhat (from 170 to 179 °C) when examining a batch of samples. The melting peak of P(3HB) is narrow and may have a small shoulder or bifurcation on the low temperature side. The enthalpy of melting varies from 70 to 120 kJ/g. The studied polymer sample had a melting enthalpy of 112 kJ/g. The crystallization temperature was 79 °C. The crystallization temperature, similar to the melting point, can vary within a fairly wide range, from 70 to 96 °C. In our experiments on isothermal crystallization, it was possible to crystallize a sample at a temperature of 130 °C. The time for the complete crystallization of the sample in this case increased from 0.46 min (crystallization at 75 °C) up to 9 min. This indicates the formation of larger crystals than during crystallization at low temperatures. The cooling rate also affects crystallization—the crystallization peak shifts towards lower temperatures with an increase in the cooling rate. The thermal stability of poly(3-hydroxybutyrate) was studied using TGA. The degradation temperature was 279 °C. The presence of a significant gap between the melting temperature and the degradation temperature is at least 100 °C. This is a positive technological property of the polymer, which makes it possible to process P(3HB) into products from the melt. Significant differences in the thermal behavior of P(3HB) in air and in an inert atmosphere (nitrogen) were not revealed.

**Figure 4:**
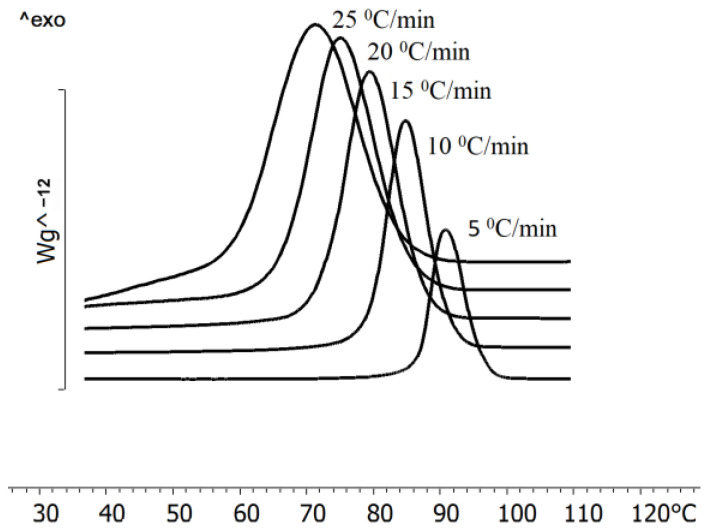
Crystallization of poly(3-hydroxybutyrate) synthesized by *Cupriavidus necator* B-10646 at different cooling rates.

The transition of polymers to a crystalline state (crystallization process) occurs during the cooling of polymer melts or during their precipitation from solutions, as well as during uniaxial tension of elastomers. Crystallization is a first-order phase transition with the values of temperature and heat of transition inherent in each polymer. The crystallization temperature of the P(3HB) samples synthesized by *C. necator* B-10646 was recorded in the range of 85–117 °C. Figure 4 illustrates the influence of the rate of cooling on the crystallization of P(3HB). The polymer crystallization peak shifts towards lower temperatures and the peak area increases with increasing the sample cooling rate. It is shown that the lower the cooling rate, the earlier the crystallization of the samples begins.

Poly(3-hydroxybutyrate), like other semi-crystalline polymers, when crystallized in the absence of a solvent or mechanical deformation, forms crystalline structures in which lamellae grow outward from the nucleation points in a circular shape until they come into contact with each other. These round structures (spherulites) are spherical in three dimensions [70,71]. Single crystals of P(3HB) are monolamellar systems; in contrast, arrays of this polymer (films, disks, etc.) are multilamellar systems that are aggregated into multioriented lamilar crystals. P(3HB) in a crystal form spherulites upon crystallization from melts to a solid state [72]. Spherulites have a semi-crystalline structure, in which highly ordered lamella plates are interrupted by amorphous regions. The size of spherulites varies widely, from micrometers to 1.0 cm, and nucleation is controlled. Severe supercooling or the deliberate addition of crystallization nuclei leads to a relatively large number of nucleation sites; in these cases the spherulites are numerous and small and interact with each other as they grow. In the case of a smaller number of nucleation centers and slower cooling, several larger spherulites are created. Nuclei can be caused by impurities, plasticizers, fillers, dyes, and other substances added to improve other polymer properties [73]. Purified PHA samples do not contain impurities that can act as nuclei for the formation and growth of spherulites. Therefore, the polymers of this family are characterized by the slow formation of large spherulites [74,75]. In the intermediate range between the two extremes, P(3HB) makes banded spherulites or double-banded spherulites which consist of alternative bright-dark-gray-dark band patterns.

Relatively recently, three new features have been established [76]: (1) the formation of spherulites with a free spiral structure, which repeats the helical structure of a natural polymer, and occurs faster than the formation of spherulites with a radial structure; (2) in the assembly of spherulite structures, the lamellae are oriented directly to the surface; and (3) material crystallized at high temperatures is less resistant to etching compared to that crystallized at low temperatures. The second and third phenomena are different from those of polymers such as polyethylene and polyethylene terephthalate, and are characteristic of extremely free volumes (spaces) in the material localized between the main assemblies of spherulites. Larger spiral spherulites in the center grow at high temperatures and are oriented in a counterclockwise direction. Small spherulites are of the opposite orientation and surround the larger ones; they were formed within two weeks of subsequent crystallization at low temperatures.

**Figure 5:**
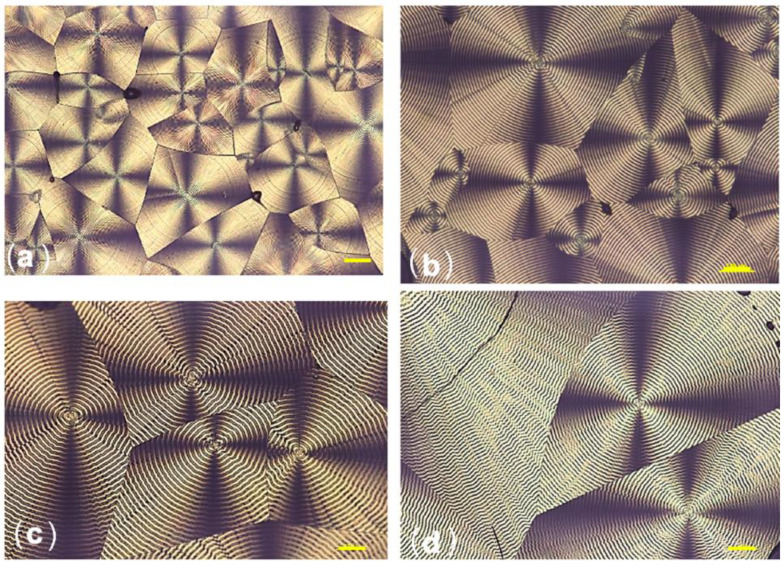
An example of the morphology of poly(3-hydroxybutyrate) spherulites during isothermal crystallization at various Tc: (**a**) 60 °C, (**b**) 70 °C, (**c**) 80 °C, and (**d**) 90 °C. Bar = 100 µm.

Understanding the morphology and crystallization behavior of PHAs is critical for controlling melt processing and product performance. The results of optical studies of spherulites formed at different temperatures of the isothermal crystallization of P(3HB) synthesized by the wild-type strain *Cupriavidus necator* B-10646 are shown in Figure 5. The structure of P(3HB) obtained using microbial fermentation is very regular.

**Figure 6:**
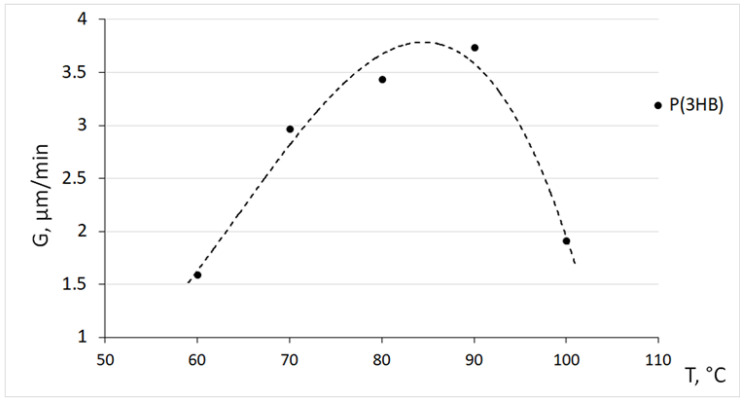
Dependence of the growth rate of spherulites (G) on the crystallization temperature for P(3HB).

It is shown that the size of the spherulites is inversely proportional to the crystallization temperature. So, at a temperature of 60 °C, the size of the spherulites varies in the range of 100–300 µm; at 80 °C—from 350 to 600 microns, and at 90 °C it reaches 1.0 mm. The radius of the spherulites increases linearly with time until the moment of impact, at any temperature. This indicates a constant growth rate of the spherulites throughout the entire process, regardless of the crystallization temperature. During isothermal crystallization of P(3HB) in a range from 60 to 90 °C, a banded structure of spherulites was observed. The distance between the bands in the spherulites increased from 1.5 to 6.0 µm with an increase in the crystallinity temperature. This result agrees with the data obtained by other authors [75]. The temperature dependence of the growth rate of spherulites (G) for a P(3HB) homopolymer sample is shown in Figure 6. The dependence curve G(T) has an extremum with a maximum Gmax at a temperature of 80–85 °C. In this region, the growth rate of spherulites is at the maximum and amounts to 3.5–3.7 µm/min. With an increase and then a decrease in temperature, the growth rate of spherulites drops sharply to a level of 1.5–1.7 µm/min.

An analysis of previous publications has shown that the process of formation and the characteristics of spherulites during PHA crystallization are most developed in relation to the P(3HB) homopolymer [77,78,79]. The dynamic growth rate of P(3HB) spherulites at different crystallization temperatures has received much attention in the literature. To understand how the nucleation process affects the crystalline morphology, two important processes and their rates should be considered. Hoffman predicted the following three—I, II, and III—crystallization regimes [80]. In [81], based on an analysis of the literature to date, data are given that show that the transitions of crystallization modes I–II and II–III have been described only for several polymers (polyethylene, low-molecular-weight isotactic polypropylene, cis-polyisoprene, and poly(3,3-dimethylthietane)). The I–II crystallization transition has been found in poly(L-lactide) and poly(1,3-dioxolane). It was shown in [65] that for P(3HB), during its crystallization at 90 °C, the growth rate of spherulites reaches 3–4 µm s^–1^, which is similar to our results. At a higher crystallization temperature, the crystallization rate is determined in region III and its values are lower [82]. It is shown that for P(3HB), the transition temperature of crystallization region II and region III is 130 °C. The process of P(3HB) crystallization in region I has not been recorded [5]. The authors of this work noted that the maximum density of the P(3HB) crystal nuclei was observed at a lower temperature range (below the crystallization temperature, at which the growth rate of spherulites is at its maximum). It was also noted that the density of the nuclei of pure P(3HB) crystals was low [83]. The maximum rate of complete crystallization for P(3HB) was determined to be in the temperature range of 60–90 °C [84].

Thus, the results obtained in the present work are consistent with similar studies performed by other authors on the study of crystallization and the formation of spherulites during the isothermal crystallization of melts of P(3HB) samples synthesized by other producers in other fermentation modes.

### 3.2. Effect of 3-Hydroxyhexanoate Monomers Incorporated into the Poly(3-Hydroxybutyrate) Chain on the Characteristics of P(3HB-co-3HHx)) Copolymers

P(3HB-*co*-3HHx) medium-chain copolymers are a particularly promising type of PHA due to their reduced crystallinity and elastomer properties [85,86]. The chemical synthesis of P(HB-*co*-HHx), its molecular structure, and mechanical properties have been reported. The thermal behavior of P(HB-*co*-HHx) has also been investigated using differential scanning calorimetry (DSC) [87,88]. Medium-chain copolymers, including P(3HB-*co*-3HHx), which have a lower melting point and reduced crystallinity, produce better polymer products, which are stronger and more elastic, and have a high elongation at break [58,87,89,90,91]. This is related to the fact that monomers of 3HHx consist of six carbon atoms, but monomers of P(3HB) consist of four carbon atoms. Since 3HHx has a longer alkyl side chain, it cannot crystallize in the 3HB lattice and hence, avoids the isodimorphism phenomenon unlike other types of MCL-PHAs, e.g., P(3HB-*co*-3HV) and P(3HB-co-4HB) copolymers, which are more amorphous in contrast to crystalline SCL-PHAs [5,92].

It has already been noted above that obtaining high yields of P(3HB-*co*-3HHx) with a high content of medium-chain monomers is a difficult task; to solve it, it is necessary to organize special biosynthesis conditions that maximize the incorporation of 3HHx monomers into the poly(3-hydroxybutyrate) chain. This requires knowledge of what type of precursor and at what concentration it should be introduced into a culture for the synthesis of PHAs. It also requires knowledge of how long after the introduction of the precursor into the medium the maximum incorporation of 3HHx monomers into the 3HB chain occurs and how to limit the endogenous degradation of 3HHx monomers along the path of β-oxidation of fatty acids [40]. An effective method for the synthesis of P(3HB-*co*-HHx) copolymers is the use of genetically modified strains [29,93,94].

Samples of the P(3HB-*co*-3HHx) copolymer with different ratios of monomers, synthesized using different technologies, and obtained from different manufacturers, were studied in this work. These are a sample from Kaneka (Japan) synthesized by the recombinant strain *Cupriavidus necator* NSDG-ΔfadB1 on soybean oil, and laboratory samples synthesized by the wild strain *C. necator* B-10646, with additions to the medium of the main carbon source (glucose or fructose), potassium hexanoate as a precursor of 3HHx, and acrylate to block the reactions of the β-oxidation cycle of fatty acids [95]. The composition and properties of the P(3HB-*co*-3HHx) samples studied, which depend on the method of synthesis and the content of the 3HHx monomers, are given in Table 1. The Kaneka sample contained low inclusions of 3HHx monomers; the laboratory samples were represented by a wider range of copolymers with a significantly higher content of 3HHx monomers in them.

A large body of data on the synthesis of P(3HB-*co*-3HHx) copolymers by various strains is given in a review [31]. The authors describe the substrates used, the obtained levels of incorporation of 3HHx monomers into the copolymer, and the physicochemical properties. In the vast majority of the papers reviewed, the content of 3HHx monomers in the copolymer did not exceed 20 mol.%; only a few of the papers discussed the inclusion of 3HHx from 32–43 to 55–70 mol.% [40,94,96]. This high result was ensured by special fermentation conditions and the involvement of genetically modified strains as producers.

**Figure 7:**
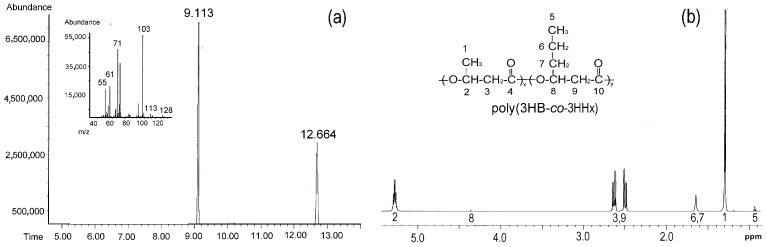
Chromatogram of the P(3HB-*co*-3HHx) sample (the retention times of the monomers, respectively, were 3HB, 9.113 min and 3HHx, 12.664 min) and mass spectrum of the methyl ester of the 3HHx monomer (**a**). The ^1^H NMR spectrum of this copolymer (**b**) synthesized by *Cupriavidus necator* B-10646. Figure 7a is taken from reference [43] with permission from Elsevier.

An ion chromatogram with mass spectrum and 1H NMR spectrum of P(3HB-*co*-3HHx) are shown in Figure 7.

Absorption bands of the asymmetric stretching vibrations of the CH_3_- and CH_2_-groups (2994, 2974, and 2936 cm^−1^), the symmetric stretching vibrations of the CH- and CH_2_-groups (2874 and 2878 cm^−1^), as well as stretching vibrations of the C=O carbonyl groups (1700 and 760 cm^−1^) were registered. The most characteristic difference of the IR spectra of the P(3HB-*co*-3HHx) specimens from those of the P(3HB) ones was the higher intensity of the absorption bands in the short-wave region of the spectrum (2800–3000 cm^−1^). The reason may be the greater contribution of the stretching vibrations of the -CH and-CH_2_-groups in P(3HB-*co*-3HHx) copolymers.

All of the samples of P(3HB-*co*-3HHx) had reduced molecular weight values and increased polydispersity values compared to the P(3HB) homopolymer (Table 1). An analysis of the influence of the 3HHx content on the molecular weight of the samples synthesized by *C. necator* B-10646 did not reveal a pronounced dependence of the weight average molecular weight on the content of 3HHx monomers in the copolymers; the M_w_ values were in the range of 486–520 kDa. The lowest weight average molecular weight (415 kDa) was determined to be for the Kaneka sample.

**Figure 8:**
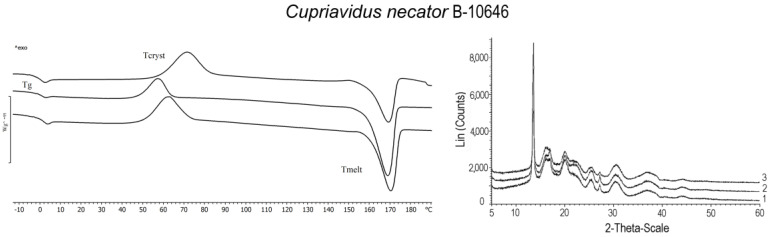

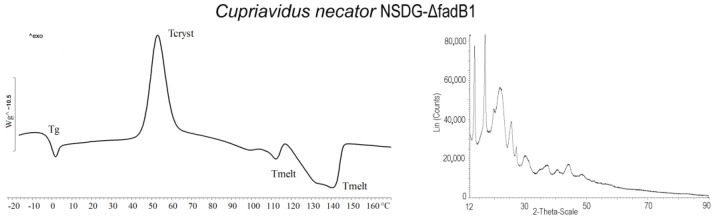
DSC and X-Ray of P(3HB-*co*-3HHx) samples synthesized by wild-type (numbering according to Table 1) and recombinant strains.

Figure 8 illustrates the temperature characteristics of the P(3HB-*co*-3HHx) samples synthesized by the wild-type strain *C. necator* B-10646. The thermal degradation temperature (T_degr_) for the copolymer samples is generally somewhat lower than that for P(3HB). Melting points were determined at 168–170 °C. The shape of the melting peaks of the copolymer is sharper than that of P(3HB); the absence of several melting peaks is shown in the melting zone. The glass transition temperature was determined to be in the range from −0.2 to −1.6 °C. A decrease in the glass transition temperature with an increase in the concentration of 3HHx monomers was noted. The crystallization temperatures of the studied samples varied depending on the content of 3HHx monomers (from 57.2 to 63.2 °C).

**Figure 9:**
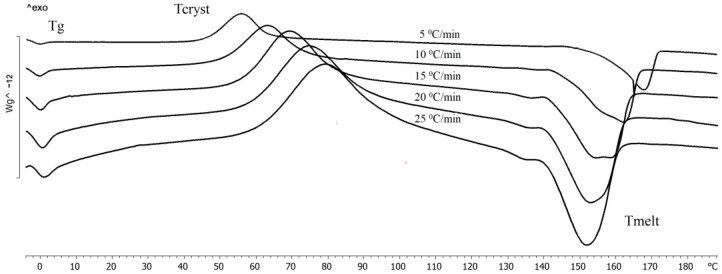
Kinetics of the crystallization of the copolymer P(HB-*co*-HHx) with a 3HHx content of 9.0 mol.%, synthesized by the wild-type strain *Cupriavidus necator* B-10646.

The crystallization kinetics of P(3HB-*co*-3HHx) at various heating and cooling rates are shown in Figure 9. At the given cooling rates, the sample does not have time to crystallize and passes into a glassy state. Upon subsequent heating, the sample devitrifies and is followed by crystallization. The crystallization peak shifts towards higher temperatures with an increase in the heating rate. The peak width also increases, in contrast to crystallization during cooling. The melting of the formed crystals occurs immediately after the end of the crystallization process at a heating rate of 25 °C/min. It should be noted that the melting peak shifted towards lower temperatures, so at a heating rate of 25 °C/min, the melting peak shifted to 152 °C.

The sample obtained with the Kaneka technology and synthesized by the recombinant strain of *C. necator* NSDG-ΔfadB1 on soybean oil had some differences (Table 1, Figure 9). The melting point was determined to be somewhat lower than that of the P(3HB) homopolymer and also of the samples of copolymers synthesized by the wild-type strain *C. necator* B-10646 on sugars. The glass transition temperature of the sample was registered as lower than that indicated by the manufacturer (2 °C) and was −2.4 °C. This was also less than the glass transition temperature recorded for the samples synthesized by a wild-type strain. The presence of two crystallization peaks, a small crystallization peak at a temperature of 69.3 °C and a crystallization peak at 53 °C, were registered on the thermogram during cooling (Figure 9) (at the values of H_crist_, respectively, of 1.84 and 40.1 J/g) and during reheating. This indicates the pre-crystallization of the sample or recrystallization due to an increase in the mobility of the amorphous part after devitrification. An analysis of the thermal stability of the Kaneka sample showed a gap between the melting point and degradation temperature of 127 °C; this is somewhat higher than that of the samples synthesized by the wild strain. The thermal degradation temperature was 268.1 °C. In the region of P(3HB-*co*-3HHx) melting, two peaks were recorded (at 112.0 and 141.0 °C) at a melting energy of 7.8 and 30.3 J/g, respectively. This is somewhat lower than that of the samples synthesized by wild strains and has, depending on the content of 3HHx monomers, one peak in this region (168.7–170.2 °C).

The thermal behavior of the P(3HB-*co*-3HHx) copolymer at room temperature was previously studied in a series of works by Japanese colleagues [63,72,97]. Their study of the crystal structure of P(HB-*co*-HHx) showed only one crystal lattice form similar to P(3HB). Using the WAXD model, it was determined that the crystal structure of P(HB-*co*-HHx) is orthorhombic, P2_1_2_1_2_1_, and has cell parameters of 5.76 Å, 13.20 Å, and 5.96 Å, which is identical to the crystal structure of poly(3-hydroxybutyrate) [30].

The data in Figure 9 and Table 1 show the significant effect of 3HHx monomers on the ratio of amorphous and crystalline phases in the P(3HB-*co*-3HHx) copolymer. Thus, in the samples synthesized by the wild-type strain *C. necator* B-10646, with an increase in the content of 3HHx from 12.0 to 31.7 mol.%, the alignment of the ratio of the amorphous and crystalline phases and a decrease in crystallinity to 50–56% were recorded. The C_x_ value of the Kaneka sample was slightly higher (60%).

The crystallization of P(HB-*co*-HHx) was studied in the temperature range from 25 to 110 °C in a recent work [30]. A WAXD diagram at room temperature shows that the P(HB-*co*-HHx) copolymer has an orthorhombic system (α = β = γ = 90°), with α = 5.76 Å, b = 13.20 Å, and c = 5.96 Å, which is identical to the crystal system of the P(3HB) homopolymer. The authors show significant changes in the lattice parameters of P(HB-*co*-HHx), with increasing temperature during crystallization, in contrast to P(3HB), in which the lattice parameters at 90 °C practically do not change, and the homopolymer retains its high crystallinity up to 140 °C. On the contrary, for P(HB-*co*-HHx), the crystallinity begins to decrease from 50 °C. Samples of P(3HB-*co*-3HHx) show recrystallization in a range from −40 to 120 °C.

**Figure 10:**
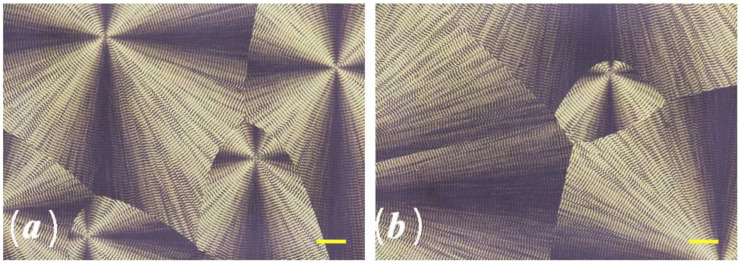
Example of the spherulite morphology of P(3HB-*co*-3HHx) copolymers synthesized by wild-type strain *Cupriavidus necator* B-10646 (**a**) and recombinant strain *Cupriavidus necator* NSDG-ΔfadB1 (**b**) at 70 °C. Bar = 100 µm.

The results of optical studies of the crystallization and formation of spherulites in the samples of P(3HB-*co*-3HHx) copolymers synthesized by the wild-type strain *C. necator* B-10646 and recombinant strain *C. necator* NSDG-ΔfadB1, with a content of 3HHx monomers, respectively, of 9.0 and 11.0 mol.%, are presented in Figure 10.

**Figure 11:**
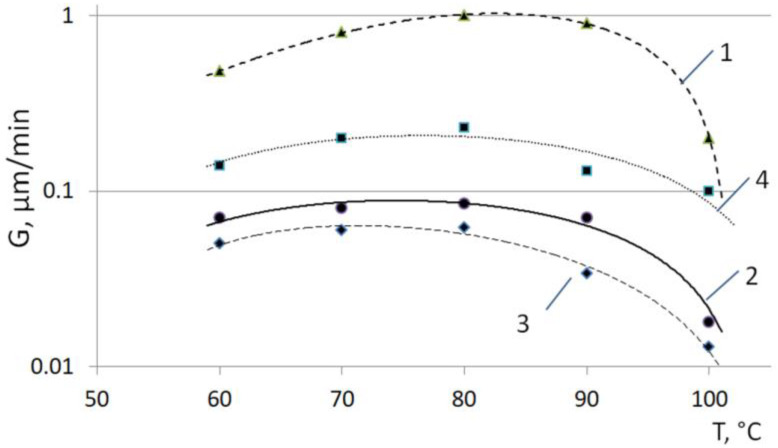
Dependence of the growth rate of spherulites (G) on the crystallization temperature for P(3HB-*co*-3HHx) copolymers synthesized by the wild-type strain *Cupriavidus necator* B-10646 (1–3, numbering according to Table 1) and recombinant strain *Cupriavidus necator* NSDG-ΔfadB1 (4).

It is shown that the direct size of spherulites in the P(3HB-*co*-3HHx) samples varies from 200 to 700 μm and can reach sizes of over 1.0 mm or more at a temperature of 90 °C, due to the low density of nucleation. At the same time, the dimensions of P(3HB-*co*-3HHx) spherulites, similarly to P(3HB), increase linearly with time until the moment of impact. The temperature dependence of the growth rate of spherulites (G) for the three samples studied, a P(3HB) homopolymer and two samples of P(3HB-*co*-3HHx) copolymers synthesized by wild and recombinant strains on various C substrates, is shown on Figure 11.

The growth rate of spherulites for P(3HB-*co*-3HHx) is much lower than that for P(3HB). Moreover, it decreases with an increase in the content of 3HHx monomers, from 9.0 to 38.0 mol.% at a given T_cryst_. The dependence curves G(T) have an extremum with a maximum Gmax at temperatures from 80 to 90 °C for all the P(3HB-*co*-3HHx) samples studied. This is analogous to the dependence of the growth rate of spherulites on the temperature determined for the P(3HB) sample. At the same time, the Gmax demonstrates a shift towards a lower T_cryst_ with an increase in the content of the 3HHx monomer in the chain of 3-hydroxybutyrate. With a change in temperature, the growth rate of spherulites in the copolymer samples containing 3HHx monomers decreases, but not as dramatically as for P(3HB).

The results obtained agree with the data of publications [81,98,99], which describe the regularities in the formation of spherulites during the isothermal crystallization of P(3HB-*co*-3HHx) copolymers. The authors of these works have shown that in P(3HB-*co*-3HHx) samples, the formation of banded spherulites similar to those obtained in this work, with a well-pronounced Maltese cross depending on the crystallization temperature of the melt, is possible. In particular, in P(3HB-*co*-3HHx) samples containing 10.0 mol.% of 3HHx monomers, the formation of banded spherulites at 85, 95, and 105 °C was shown. However, with a further increase in T_cryst_ to 115 °C, banded spherulites were not observed. The authors of the review [73] collected the known data on the morphological features of spherulites formed during the crystallization of composites based on PHAs, as well as PHAs of various chemical compositions. They also described that, depending on the type of polymers and crystallization conditions, the formation of both homogeneous spherulites (without bands) and banded spherulites (ring-shaped) with alternating light and dark bands is possible. Generalized data on the effect of PHA composition on the radial growth rate of spherulites during the isothermal crystallization of polymers are generally consistent with the results of the presented work, which show a higher growth rate of spherulites for P(3HB) compared to P(3HB-*co*-3HHx), and the influence of the content of 3HHx monomers and temperature for this indicator.

## 4. Conclusions

Samples of the medium-chain copolymer P(3HB-*co*-3HHx), synthesized with different technologies and containing various inclusions of 3HHx monomers (from 9.0 to 38.0 mol.%), were studied in comparison to the P(3HB) homopolymer. Laboratory samples synthesized by the wild-type strain *C. necator* B-10646 on sugars, and an industrial sample manufactured by Kaneka, synthesized by the recombinant strain *C. necator* NSDG-ΔfadB1 on soybean oil, were studied. All the copolymer samples, regardless of the synthesis conditions and the ratio of monomers, had lower weight average molecular weights (415–520 kDa) and increased polydispersity values (2.8–4.3) compared to the P(3HB) homopolymer (720 kDa and 2.2). The 3-hydroxyhexanoate monomers, when the highly crystalline (70–76%) 3-hydroxybutyrate was included in the chain, had a significant effect on the ratio of ordered and disordered phases in the polymer, and caused an increase in amorphization and a decrease in the degree of crystallinity to 49–50% in the samples synthesized by a wild strain, and up to 60% in the case of the recombinant sample. Some differences between the industrial sample P(3HB-co-3HHx) and the laboratory ones were found in the study of temperature characteristics. The industrial sample had a lower glass transition temperature (−2.4 °C), two peaks in the crystallization and melting regions, a lower melting point (112/141 °C), and a more pronounced gap between T_melt_ and T_degr_ compared to the laboratory ones. The process, shape, and size of spherulites formed during the isothermal crystallization of P(3HB) and P(3HB-*co*-3HHx) were generally similar. However, the maximum growth rate of spherulites recorded for all the samples at 85–90 °C for the copolymer was significantly lower (0.06–1.25 µm/min) than that for P(3HB) (3.5–3.7 µm/min), and was affected by the content of 3HHx monomers. In general, and in connection with the increasing role of P(3HB-*co*-3HHx) copolymers as a new, technologically advanced “green” bioplastic, the study of the thermal properties and crystallization mechanisms of these copolymers synthesized by various strains on various substrates is important for improving the processing regime and technologies for specialized products in manufacturing processes.

## Figures and Tables

**Table 1:** Composition and physicochemical properties of P(3HB-*co*-3HHx).

Sample	PHA Composition, mol.%		M_w_, kDa	Ð	C_x_, %	T_g_, °C	T_cryst_, °C	T_melt_, °C	T_degr_, °C
The sample was synthesized by *Cupriavidus necator* NSDG-ΔfadB1 from Kaneka (Japan)									
1	3HB	3HHx	415	2.8	60	−2.4	69.3/ 53.0	112/ 141	268.1
	89.0	11.0							
The samples were synthesized by *Cupriavidus necator* B-10646 [41,96]									
1	3HB	3HHx							
	91.0	9.0	520	3.9	60	−0.2	63.2	170.2	262.7
2	83.6	16.4	390	4.3	49	−0.6	57.2	168.7	281.5
3	62.0	38.0	486	3.7	52	−1.6	71.2	169.2	260.1

## Data Availability

All data are available in the paper.
